# Relationship between Aflatoxin Contamination and Physiological Responses of Corn Plants under Drought and Heat Stress

**DOI:** 10.3390/toxins4111385

**Published:** 2012-11-20

**Authors:** Hirut Kebede, Hamed K. Abbas, Daniel K. Fisher, Nacer Bellaloui

**Affiliations:** 1 Crop Genetics Research Unit, Agricultural Research Service, United States Department of Agriculture, 141 Experiment Station Road, Stoneville, MS 38776, USA; Email: nacer.bellaloui@ars.usda.gov; 2 Biological Control of Pests Research Unit, Agricultural Research Service, United States Department of Agriculture, 141 Experiment Station Road, Stoneville, MS 38776, USA; Email: hamed.abbas@ars.usda.gov; 3 Crop Production Systems Research Unit, Agricultural Research Service, United States Department of Agriculture, 141 Experiment Station Road, Stoneville, MS 38776, USA; Email: daniel.fisher@ars.usda.gov

**Keywords:** aflatoxin contamination, *Aspergillus flavus*, cell membrane thermostability, drought stress, heat stress, oxidative stress, photosynthetic pigments, Photosystem II

## Abstract

Increased aflatoxin contamination in corn by the fungus *Aspergillus flavus* is associated with frequent periods of drought and heat stress during the reproductive stages of the plants. The objective of this study was to evaluate the relationship between aflatoxin contamination and physiological responses of corn plants under drought and heat stress. The study was conducted in Stoneville, MS, USA under irrigated and non-irrigated conditions. Five commercial hybrids, P31G70, P33F87, P32B34, P31B13 and DKC63-42 and two inbred germplasm lines, PI 639055 and PI 489361, were evaluated. The plants were inoculated with *Aspergillus flavus* (K-54) at mid-silk stage, and aflatoxin contamination was determined on the kernels at harvest. Several physiological measurements which are indicators of stress response were determined. The results suggested that PI 639055, PI 489361 and hybrid DKC63-42 were more sensitive to drought and high temperature stress in the non-irrigated plots and P31G70 was the most tolerant among all the genotypes. Aflatoxin contamination was the highest in DKC63-42 and PI 489361 but significantly lower in P31G70. However, PI 639055, which is an aflatoxin resistant germplasm, had the lowest aflatoxin contamination, even though it was one of the most stressed genotypes. Possible reasons for these differences are discussed. These results suggested that the physiological responses were associated with the level of aflatoxin contamination in all the genotypes, except PI 639055. These and other physiological responses related to stress may help examine differences among corn genotypes in aflatoxin contamination.

## 1. Introduction

Corn production in the southern United States frequently encounters a period of drought and heat stress during flowering and kernel development. These weather conditions have been reported to be the major factors in increased aflatoxin contamination produced by the fungi *Aspergillus flavus* and *A. parasiticus* in corn and other economically important crops such as peanuts, cotton and tree nuts [1,2]. *A. flavus *infection and subsequent aflatoxin contamination is a serious issue in the southern US. Aflatoxins are toxic, highly carcinogenic secondary metabolites of these pathogens, which when produced during fungal infection of a susceptible crop in the field or after harvest, contaminate food and feed and threaten human and animal health [1,2]. 

Previous studies have shown that reduction in drought stress by irrigation reduces aflatoxin contamination, and drought stress tolerant corn varieties produce significantly less aflatoxin in the field in drought stress conditions [3,4,5]. Under conditions of drought and high temperatures, lowering soil temperature by irrigation was also found to reduce aflatoxin contamination [3,6]. In addition to drought and heat stress, other factors that produce stress on the plants such as inadequate plant nutrition, insect feeding on developing kernels, weed competition, excessive plant density, plant diseases, and other biotic and abiotic stresses facilitate the infection and production of aflatoxin by the fungus [2,7,8]. Several studies have shown that aflatoxin production is associated with oxidative stress, which is caused by abiotic and biotic stresses on plants [9,10,11,12,13,14]. Effects of drought, heat and other stress factors on physiological traits such as photosynthetic pigments, cell membrane thermostability, and maximum quantum efficiency of photosystem II are associated with oxidative stress [15,16,17,18,19,20,21]. The aim of this study was to evaluate the relationship between aflatoxin contamination and these stress response-related physiological traits in corn genotypes under drought and heat stress. Moisture and temperature conditions in the soil and the air were monitored to determine the extent of stress imposed on the plants. 

## 2. Results

### 2.1. Environmental Moisture and Temperature Conditions

The 2010 growing season was hotter and drier than that of 2009, with temperatures of 36 °C and 33 °C averaged over the months of June, July and August for 2010 and 2009, respectively, and a growing season total precipitation of 266 mm and 666 mm for 2010 and 2009, respectively (Mississippi State University weather network). In addition to the weather information, data from the plot-level automated monitoring system was used to observe the level of drought and heat stress imposed on the corn plants. Soil moisture in the non-irrigated plots was about four times lower than in the irrigated plots, and air temperature in the canopy microclimate and soil temperature in each plot were 2 °C–5 °C higher in the non-irrigated plots than in the irrigated plots [22]. Differences were observed among the plots of each corn genotype in these temperature and moisture measurements. Details are presented in Kebede *et al.* [22]. These data were important in explaining the effect of the temperature and moisture conditions on the growth and development of the corn plants as well as the fungus.

### 2.2. Aflatoxin Contamination

**Figure 1:**
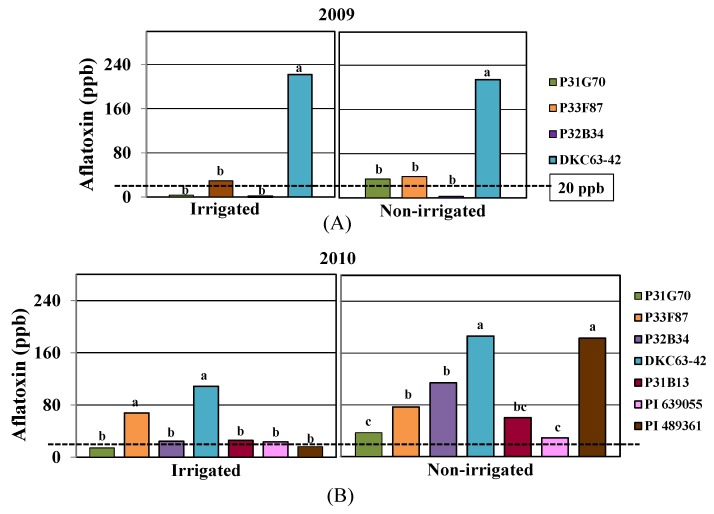
Aflatoxin contamination in corn kernels under irrigated and non-irrigated conditions in (**A**) 2009 and (**B**) 2010. Different letters on top of bars show significant differences (*p* < 0.05). The dashed lines in the graph (20 parts per billion, ppb) show the allowable level of aflatoxin contamination by the US Food and Drug Administration (FDA) [23].

Aflatoxin contamination in the corn kernels was higher in 2010 than in 2009 under both soil moisture treatments (Figure 1), with levels significantly higher (*p* < 0.046) in the non-irrigated than the irrigated plots in 2010. In 2009, aflatoxin was determined only on four of the corn genotypes (P31G70, P32B34, P33F87, and DKC63-42) due to glyphosate damage on P31B13, PI 639055 and PI 489361 plants. Significant differences were observed among the genotypes in aflatoxin contamination in both years. In 2009, DKC63-42 had more than a nine-fold aflatoxin contamination compared to the other three hybrids, while P32B34 had the least amount under both soil moisture treatments (Figure 1A). The aflatoxin levels in P32B34 and P31G70 in the irrigated treatments, and in P32B34 in the non-irrigated treatments, were below 20 parts per billion (ppb), which is the allowable level of aflatoxin set by the FDA in corn grain that is used for human consumption [23]. In 2010, the aflatoxin test included the genotypes which were not tested in 2009 (P31B13, PI 639055 and PI 489361). In the irrigated treatments, aflatoxin was around the 20 ppb threshold level for all genotypes except for two hybrids, P33F87 (68 ppb) and DKC63-42 (109 ppb), which were significantly higher (Figure 1B). The aflatoxin resistant line (PI 639055) and P31G70 had levels below the threshold, 16 and 14 ppb, respectively. However, under non-irrigated treatments, all genotypes had levels above 20 ppb including the aflatoxin resistant line. The resistant line had the lowest contamination (29 ppb) followed by P31G70 (33 ppb), but there was no significant difference between the two values. DKC63-42 and PI 489361 had the highest level of aflatoxin among all genotypes, 186 and 185 ppb, respectively, which were more than double the accumulation of aflatoxin in most of the other genotypes. Aflatoxin contamination was not significantly different among P33F87, P32B34, and P31B13 under non-irrigated conditions, with intermediate levels compared to the other genotypes.

### 2.3. Physiological Responses to Drought and Heat Stress

#### 2.3.1. Leaf Water Potential and Canopy Temperature

**Figure 2:**
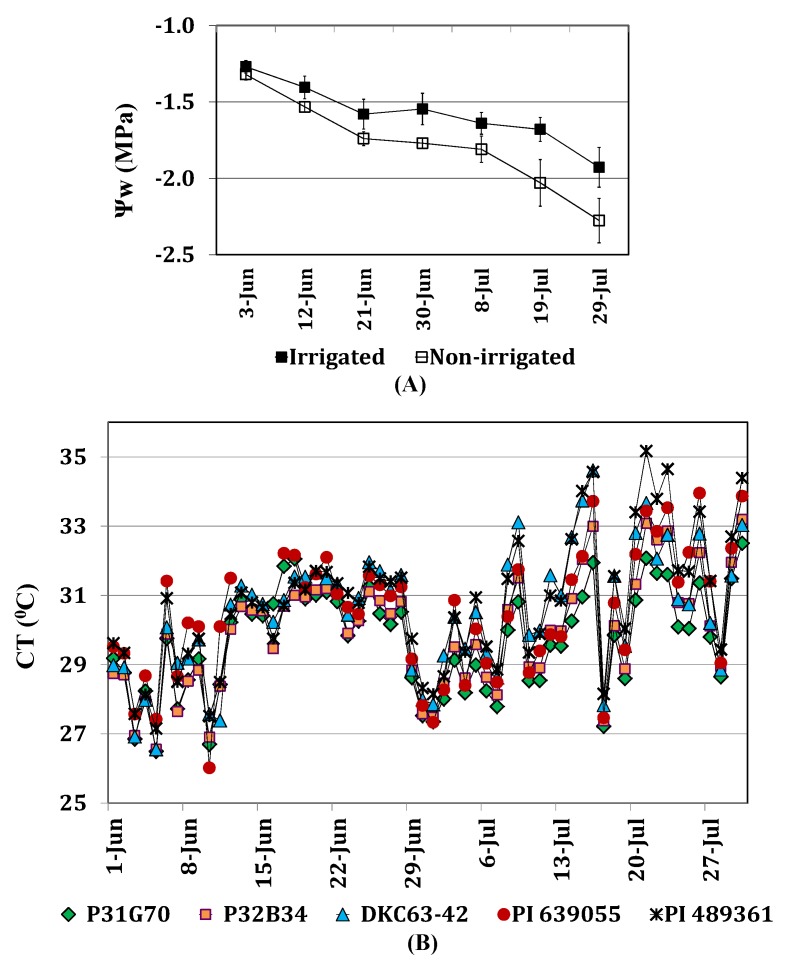
Leaf water potential (Ψ_w_) and canopy temperature (CT) for hybrids P31G70, P32B34, DKC63-42 and inbred lines PI 639055 and PI 489361 under irrigated and non-irrigated treatments in the months of June and July, 2010; (**A**) mean values across genotypes for Ψ_w_ on seven sampling dates; (**B**) mean values for hourly measurements (hours between 13:00 and 17:00) of CT in the non-irrigated plots of each genotype in 2010.

Leaf water potential (Ψ_w_) and canopy temperature (CT) were used to assess moisture deficit and heat stress effects on the plants. Leaf water potential was negatively correlated with CT (*r* = −0.4907; *p* < 0.0516) and positively correlated with soil water potential (*r* = 0.6550; *p* < 0.0080), and it was significantly lower in the non-irrigated than the irrigated plants in both years, as shown in Figure 2A. Leaf water potential values were lower than −1.5 MPa after the second sampling date (June 12) in both irrigated and non-irrigated plants, but it was significantly lower in the non-irrigated plots going down as low as −2.3 MPa. No significant differences were detected among the corn genotypes in Ψ_w_ due to large variability in values within each genotype. Canopy temperature in the non-irrigated plots was 2 °C–5 °C higher than in the irrigated plots [22]. Differences were observed in CT among the corn genotypes. Figure 2B shows CT among the genotypes under non-irrigated conditions in 2010. Among the hybrids, DKC63-42 had the highest CT, particularly after the middle of June, followed by P32B34. The two germplasm lines, PI 639055 and PI 489361, had higher CT. Hybrid P31G70 had the lowest CT among all the genotypes. 

#### 2.3.2. Photosynthetic Pigments

**Figure 3:**
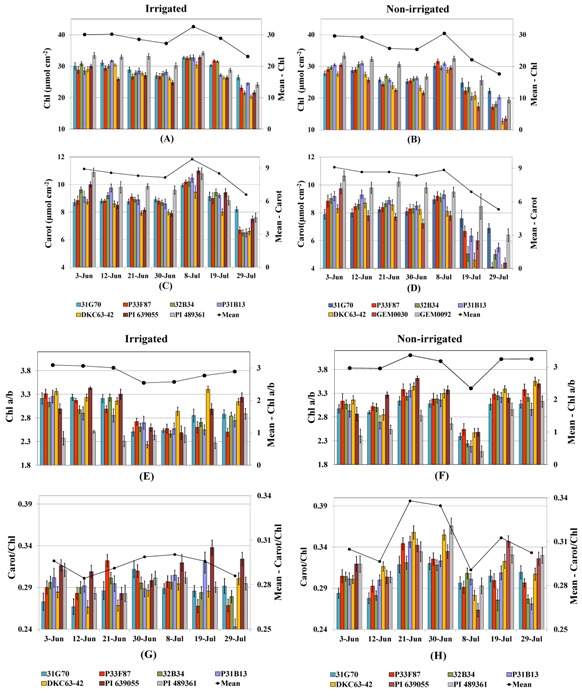
Photosynthetic pigment content in leaves of seven corn genotypes sampled in June and July, 2010, under irrigated and non-irrigated treatments. Values for individual genotypes are shown in bar graphs and mean values for each sampling date are shown in line graph on top of each bar graph (secondary axis): (**A** and **B**) chlorophyll (Chl); (**C** and **D**) carotenoids (Carot); (**E** and **F**) chlorophyll a to chlorophyll b ratio (Chl a/b); (**G** and **H**) carotenoids to chlorophyll ratio (Carot/Chl). Samples were taken from eight plants for each genotype.

Changes in chlorophyll and carotenoid content followed a similar pattern to the changes in Ψ_w_. Highly significant correlation was observed between Ψ_w_ and the photosynthetic pigments (chlorophyll, *r* = 0.8532, *p* < 0.0012; carotenoids, *r* = 0.8821, *p* < 0.0007). Irrigation started in early June and drought stress started showing effect on the samples taken on June 21 and continued through the end of July. Mean values for chlorophyll and carotenoid contents were significantly higher in irrigated plants compared to the non-irrigated plants on these sampling dates (Line graphs in Figure 3A–D). However, reductions in these pigments were also observed in samples from the irrigated plants. Air temperature was high during this period of time, with average maximum temperatures in the range of 36 °C–38 °C during June 13–29 [22]. An increase in these photosynthetic pigments in the July 8 sampling date was due to several rain showers at the end of June and beginning of July with slightly cooler temperatures. In addition, it could be due to the age of the leaves, older leaves gradually accumulating more chlorophyll. Chlorophyll a to b ratio (Chl a/b) (Figure 3E,F) and carotenoids to chlorophyll ratio (Carot/Chl) (Figure 3G,H) were significantly higher in the non-irrigated plants.

Statistically significant differences were observed among the genotypes in these photosynthetic pigments as shown in Figure 3 (Bar graph). The germplasm line PI 489361 had significantly higher chlorophyll and carotenoids among all the genotypes (*p* < 0.0001) under both soil moisture treatments, which could be attributed to its thicker leaf [22]. PI 639055 had significantly lower chlorophyll and carotenoids than the other genotypes in most of the sampling dates under non-irrigated conditions, but had higher Chl a/b ratios. Among the hybrids, DKC63-42 had lower chlorophyll under non-irrigated conditions, and lower content of carotenoids under irrigated conditions. DKC63-42 had higher Chl a/b than the other genotypes under both irrigated and non-irrigated treatments (Figure 3E,F). PI 489361 had the lowest Chl a/b ratio, but this has been observed under non-stress conditions in other experiments under greenhouse and field conditions, which indicates that this ratio is not related to the plant's response to the stress conditions. Carotenoids to chlorophyll ratios were not consistent for all genotypes across sampling dates under both soil moisture treatments, but PI 639055, PI 489361 and DKC63-42 had higher ratios on several of the sampling dates (Figure 3G,H).

#### 2.3.3. Chlorophyll Fluorescence and Cell Membrane Thermostability

**Figure 4:**
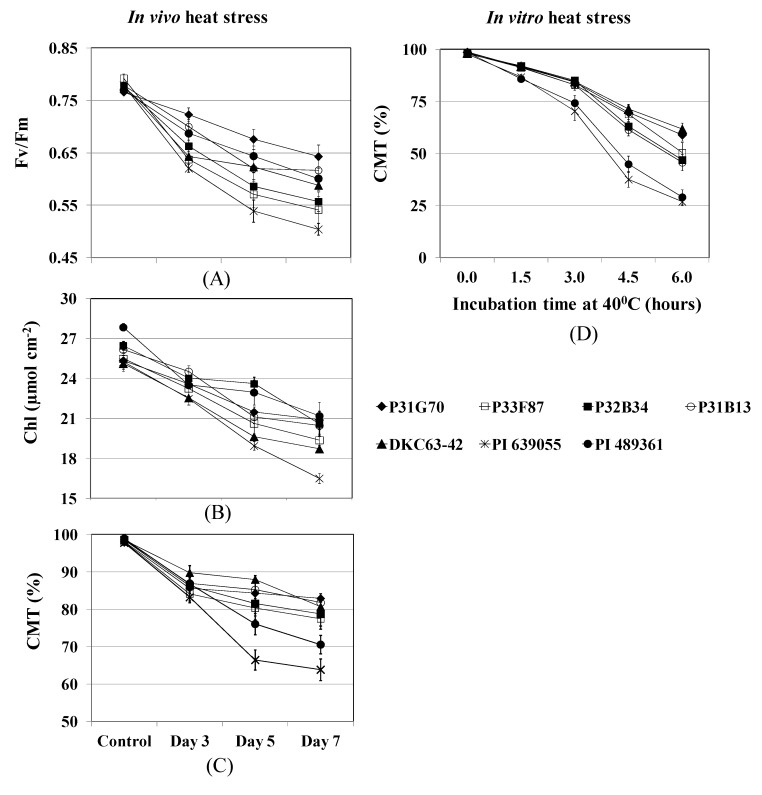
*In vivo *heat stress effects (38 °C/33 °C day/night temperature for 7 days) in seven corn genotypes on (**A**) maximum quantum efficiency of Photosystem II (Fv/Fm); (**B**) chlorophyll content (Chl) and (**C**) cell membrane thermostability (CMT) on six-week old plants under greenhouse conditions; the same heat-stressed leaves were used for Fv/Fm, Chlorophyll and CMT determination; (**D**) *In vitro* heat stress effects on CMT in leaf discs collected from non-stressed field-grown plants and incubated in deionized water at 40 °C for 6 h. Measurements were made every 1.5 h.

Maximum quantum efficiency of PS II (Fv/Fm), Chlorophyll content, and CMT were significantly reduced when heat stress was imposed on the corn plants *in vivo *at 38 °C/33 °C (day/night temperature) for seven days under greenhouse conditions (Figure 4A–C). Significant differences were observed among genotypes in these parameters. A gradual reduction was observed in Fv/Fm during the course of the seven-day heat treatment: P31G70 had the highest and PI 639055 had the lowest Fv/Fm throughout the treatment with significant differences among the genotypes (Figure 4A). The greatest reduction in chlorophyll was observed in PI 639055 by the end of the heat stress treatment (Figure 4B). Reduction in chlorophyll in PI 489361 was comparable to that of the commercial hybrids because of the higher content due to its thicker leaves as mentioned above, which also helped it to have a comparable Fv/Fm to that of the commercial hybrids. Cell membrane injury was significantly lower in the commercial hybrids than in the germplasm lines (Figure 4C). After five days of treatment, PI 639055 and PI 489361 had drastic reductions in CMT and had significantly lower CMT than the commercial hybrids. There were also differences among the hybrids: DKC63-42 had higher CMT on day 3 and day 5 than the other hybrids, however, P31G70 and P31B13 had a more gradual reduction in CMT and, by the end of the 7-day heat stress treatment, P31G70 had the highest CMT even though it was not significantly different from the other two genotypes. Similar results were observed in *in vitro* heat stress treatments by exposing leaf discs of field grown plants (which were not exposed to heat or drought stress) for 6 h at 40 °C (Figure 4D). In this treatment, the germplasm lines, particularly PI 639055, reached 50% relative injury in about 4 h, whereas it took about 6 h for the commercial hybrids to reach that level of injury.

#### 2.3.4. Seed Composition

In the commercial hybrids, soil moisture treatments had significant effect on kernel protein and starch percentages (*p* < 0.0019 and *p* < 0.0006, respectively); kernels from non-irrigated plots had higher protein percentage, and those from irrigated plots had higher starch percentage (Table 1). However, in DKC63-42, both protein and starch percentages did not show differences between the two soil moisture treatments and protein percentages were significantly higher in DKC63-42 than in the other commercial hybrids under both soil moisture treatments. Inbred lines PI 639055 and PI 489361 had protein percentages similar to that of DKC63-42 in both soil moisture treatments. Hundred kernel weight was significantly lower in these three genotypes, which may partly explain the higher protein content in these genotypes. No significant differences were observed among genotypes in fiber and oil percentage in the kernels.

**Table 1:** Protein and starch concentrations (in percentage) in the kernels and kernel weight in the corn genotypes under irrigated and non-irrigated treatments in 2009 and 2010.

Genotype	Protein (%)		Starch (%)		100 kernel wt (g)	
	Irrigated	Non-irrigated	Irrigated	Non-irrigated	Irrigated	Non-irrigated
**2009**						
P31G70	7.5 ^c^	8.1 ^b^	63.3 ^b^	62.4 ^b^	28.8 ^b^	28.7 ^a^
P33F87	7.4 ^c^	8.0 ^b^	64.5 ^a^	63.3 ^a^	30.2 ^a^	29.6 ^a^
P32B34	8.1 ^b^	8.3 ^b^	63.4 ^b^	62.5 ^b^	29.5 ^a^	30.0 ^a^
DKC63-42	9.0 ^a^	9.0 ^a^	62.7 ^c^	62.6 ^b^	30.2 ^a^	29.0 ^a^
**2010**						
P31G70	7.9 ^b^	8.8 ^a^	63.0 ^b^	62.6 ^b^	28.5 ^b^	27.9 ^b^
P33F87	7.5 ^c^	8.2 ^b^	65.1 ^a^	62.5 ^b^	31.5 ^a^	30.9 ^a^
P32B34	7.9 ^b^	7.8 ^b^	63.1 ^b^	62.7 ^b^	32.2 ^a^	29.1 ^a^
P31B13	7.6 ^b^	8.1 ^b^	62.7 ^b^	62.0 ^b^	31.0 ^a^	29.1 ^a^
DKC63-42	9.2 ^a^	9.0 ^a^	63.3 ^b^	62.8 ^a^	29.8 ^b^	27.5 ^b^
PI 639055	9.3 ^a^	9.2 ^a^	61.8 ^b^	62.7 ^b^	29.6 ^b^	28.7 ^b^
PI 489361	8.5 ^a^^b^	8.9 ^a^	62.5 ^b^	63.2 ^a^	30.0 ^b^	28.5 ^b^

Means with different letters in columns are significantly different (*p * < 0.05).

## 3. Discussion

Previous studies have shown that aflatoxin contamination was higher in kernels from non-irrigated corn plants than those from irrigated plants under drought stress conditions [3,5,24]. Similarly, in this study, there was a significant increase in aflatoxin contamination in 2010 and a slight increase in 2009 in the kernels of the non-irrigated plants. The effect of drought stress on aflatoxin contamination, however, was confounded by heat stress. Data from the microcontroller-based monitoring system and weather information showed that the corn plants were exposed to moisture deficit and high air and soil temperatures after flowering in the non-irrigated treatments, particularly in 2010, which resulted in significant reduction in leaf water potential and photosynthetic pigments in 2010 compared to 2009 [22]. This may explain the difference in aflatoxin contamination between the two years. Differences in soil moisture, soil temperature, and the air temperature in the canopy micro-climate in the plots of the individual genotypes were reflected in the physiological response differences among the genotypes [22].

Photosynthetic pigment content, Fv/Fm, CMT and CT, showed that the corn genotypes responded differently to drought and heat stress. Some morphological traits may also have contributed to the differences among the genotypes [22]. Reductions in chlorophyll and carotenoids were observed in the plants under both irrigated and non-irrigated treatments. Reduction in these photosynthetic pigments in the irrigated plots could be due to heat stress and its indirect effect on Ψ_w_ as discussed in detail in Kebede *et al*. [22]. However, in the non-irrigated plants, reductions were greater due to a combination of drought and heat stress. Earlier studies in corn have shown that large water deficits and heat stress cause loss of chloroplast membrane integrity and damage to the photosynthetic apparatus resulting in reduction in photosynthetic pigments [17,18,21]. Thus, reductions in chlorophyll and carotenoids in the stressed corn plants could have been caused by degradation of these pigments or inhibition of their biosynthesis due to damage to the photosynthetic apparatus. Elevated Chl a/b and Carot/Chl ratios are good indicators of the level of stress on a plant [18,22]. Mean values for these two parameters in the non-irrigated plants were significantly higher than in the irrigated ones indicating that the non-irrigated plants were under greater stress. 

Results from the greenhouse experiment showed that heat stress reduced Fv/Fm, which is a measure of the maximum quantum efficiency of PS II, suggesting that a structural and functional disorder of the photosynthetic apparatus and damage to the PSІІ had occurred [17,18]. Under high temperatures, peroxidation of unsaturated fatty acids in membrane lipids is responsible for cell membrane damage [18,21,25]. Results in this study showed that heat stress significantly decreased CMT in the corn plants, suggesting that damage to the cell membranes may have been caused by lipid peroxidation. Lower chlorophyll and carotenoid content, and higher Chl a/b and Carot/Chl ratios in DKC63-42 and PI 639055, reduced CMT in PI 639055 and PI 489361, and the lowest Fv/Fm in PI 639055 indicated that these genotypes had more damage under drought and heat stress compared to the other genotypes. The higher content in chlorophyll and carotenoids in PI 489361 is due to its thicker leaves which contain more photosynthetic apparatus than the other genotypes [22], and so does not suggest that it was tolerant to a combination of these stresses. 

Earlier studies have shown that stress tolerant corn genotypes produce significantly less aflatoxin [4,5]. Aflatoxin contamination was significantly lower in the drought and heat stress tolerant genotype, P31G70, but two of the three stress sensitive genotypes, DKC63-42 and PI 489361, had significantly higher contamination under non-irrigated conditions. Hybrid P31G70 had the highest Fv/Fm and CMT values among the genotypes at the end of the heat stress treatment, which may suggest that there was less damage to the PSII system and its cell membranes. Furthermore, product ratings for P31G70 show that it has a maximum score for drought stress tolerance as well as for the stay-green trait [26]. Stay-green, also called delayed leaf senescence, is a trait that resists premature death from unidentified causes [27]. During post-flowering drought-stress, stay-green lines resist premature plant and leaf death and extend the period of active assimilation past physiological maturity [28,29]. P31G70 showed higher chlorophyll content towards the end of grain fill stage, indicating that senescence may have been delayed in this hybrid. The stay-green trait is associated with higher leaf water content [28,29,30], and in the current study, P31G70 had the lowest canopy temperature of all the genotypes, which could be due to a higher leaf water content that helped it cool its leaves and reduce temperatures in the canopy microclimate and the soil. These factors may have helped the plant to be under less stress than the other genotypes and contribute in the reduction of aflatoxin production. Moreover, reduced day and night soil temperatures in the plots of this genotype might have created less favorable condition for fungal growth compared to the situation for the other genotypes, as earlier reports showed that high soil temperature, particularly night temperature, favors *A. flavus* growth [3,6,31]. 

Hybrid DKC63-42 had the highest aflatoxin contamination under both irrigated and non-irrigated conditions in both years. The increased aflatoxin contamination in the irrigated plants could not have been due to heat stress alone because *in vivo* and *in vitro* heat stress treatments showed that its values for CMT and Fv/Fm were comparable to those of the other commercial hybrids. Moreover, its kernel protein percentages were significantly higher than those of the other commercial hybrids under both soil moisture treatments in both years and soil moisture treatments didn't affect its protein percentage. Therefore, in addition to drought and heat stress, there may be other factors that contributed to the increased aflatoxin contamination in this hybrid. No direct relationship was observed between aflatoxin contamination and seed composition in this study. 

Inbred PI 489361 was released as a drought stress tolerant line and measurements on its other physiological and morphological characteristics suggested that its major drought stress tolerance mechanism is water use efficiency (data not shown). However, it was sensitive to heat, as indicated by its low CMT under *in vivo* and *in vitro* heat stress treatments. Under field conditions, a combination of drought and heat stress, and high soil temperatures, could have contributed to the high production of aflatoxin in PI 489361. 

The aflatoxin-resistant inbred germplasm, PI 639055, even though it was the most sensitive genotype to drought and heat stress, had the lowest aflatoxin contamination. This is contrary to what was discussed above. Hence, attempt was made to suggest possible reasons behind this scenario. In aflatoxin resistant lines, several corn kernel proteins with antifungal activities have been associated with their resistance to and inhibition of fungal growth or aflatoxin biosynthesis by *A. flavus*, which include proteins such as a 14-kDa trypsin inhibitor, chitinase, pathogenesis-related protein, (ZmPR-10), and a lectin-like protein, (ZmCORp) [8,32,33,34,35]. Kernel pericarp wax characteristics have also been indicated as a source of resistance to fungal infection [36,37,38]. But, most recent studies have reported that kernel resistance may require not only the presence of high levels of antifungal proteins, but also high levels of stress-related proteins which also enhance disease resistance [5,8,39,40,41]. In this study, PI 639055 may have these antifungal and stress-related proteins that could have minimized infection by *A. flavus* and aflatoxin production, regardless of the plants being under greater stress. However, antioxidative proteins, which are part of the stress-related proteins [42,43], may not have been the major players in supporting aflatoxin resistance in PI 639055 because the physiological measurements suggested that this genotype had more damage to its photosynthetic apparatus and cell membranes than in the other genotypes under drought and heat stress. This may show the complexity behind aflatoxin resistance in this genotype. 

There is evidence that oxidative stress, which is caused by both abiotic and biotic stresses on plants, triggers aflatoxin production by the fungus, and it could be a means by which the fungus protects itself from oxidative stress [9,10,11,12,13,14]. Many studies have shown that reductions in the physiological response traits measured in this study (photosynthetic pigment content, Fv/Fm and CMT) under drought and heat stress are caused by oxidative stress [15,16,17,18,19,20,21]. Oxidative stress is a state in which plant cells are damaged by excessive generation of reactive oxygen species (ROS) due to stress [15,16,17]. Chloroplasts are a major source of ROS and enhanced production of ROS due to stress causes oxidative damage to the photosynthetic apparatus, resulting in a decline in photosynthetic pigments [17,18,19,20,21]. Oxidative stress also brings about peroxidation of unsaturated fatty acids in membrane lipids and is responsible for cell membrane damage [18,21,25], as shown in a decline in CMT in this study. Thus, oxidative stress could be the link between aflatoxin contamination and these physiological responses in the corn plants under drought and heat stress. However, this assumption has yet to be investigated to confirm the relationship.

## 4. Materials and Methods

### 4.1. Plant Material, and Moisture and Temperature Measurements

Five commercial corn hybrids and two corn inbred germplasm lines from the Germplasm Enhancement of Maize (GEM) Program, USDA-ARS, Ames, IA, USA, (Table 2) were grown under two soil moisture treatments, irrigated and non-irrigated, in 2009 and 2010 in Stoneville, MS [22]. The experimental design was a split-plot with soil moisture treatment as main plot and corn genotype as subplot with four replicates, and both main and subplots were randomized. Treatments were arranged in plots each consisting of four rows 9.1 m long, with 1 m row spacing, and planted at a seeding rate of about 70,000 plants/ha. In order to avoid moisture diffusion to the non-irrigated plots, non-irrigated buffer strips consisting of four rows were planted between and parallel to all irrigated and non-irrigated treatments. Prior to planting, K as muriate of potash at a rate of 67 kg/ha and N as NH_4_NO_3_ at a rate of 112 kg/ha were applied and incorporated into the soil. Additional N in a liquid fertilizer form at 100 kg/ha was applied at growth stage V6. Irrigation treatments were applied beginning at anthesis (early June), using furrow irrigation and a schedule commonly used for corn production in the region, which is a 10-day rotation between irrigations or after a rain event of 25 mm or more after silking.

**Table 2:** Commercial corn hybrids and inbred germplasm lines used in the study.

Name	Type	Trait
P31G70	commercial hybrid	drought stress tolerant + stay-green
P33F87	commercial hybrid	drought stress tolerant
P32B34	commercial hybrid	needs irrigation for good performance
P31B13	commercial hybrid	corn borer resistant (Bt)
DKC63-42	commercial hybrid	aflatoxin susceptible
PI 639055 (GEMS-0030)	Inbred germplasm (tropical background)	aflatoxin resistant
PI 489361 (GEMS-0092)	Inbred germplasm (tropical background)	drought stress tolerant

Canopy, air, and soil temperatures and soil moisture status were collected hourly in each plot throughout the cropping season using automated microcontroller-based data logging systems as described in detail by Fisher and Kebede [44]. The study was conducted under optimum crop management practices, except for the non-irrigated treatment which was intended to impose drought stress on the plants. 

### 4.2. Fungal Inoculation and Aflatoxin Analysis

*Aspergillus flavus *(strain K54) inoculum was prepared according to Abbas *et al*. [45], and wheat grain was used as the inoculant carrier. Inoculations were made at mid-silk stage of the corn plants by scattering the treated wheat seed by hand in the middle two furrows of the four-row corn plots at a rate of 20 kg ha^−1^. At the end of the season, the two middle rows of the corn plots of each genotype were harvested and a representative sample of the kernels was collected. Grain samples of at least 1 kg were ground using a Romer mill (Union, MO, USA) and 20 g of the ground sample were used for aflatoxin analysis. Extraction and clean-up of aflatoxins from corn samples and aflatoxin determination were carried out as described by Abbas *et al*. [46]. 

### 4.3. Physiological Measurements

#### 4.3.1. Plant Water Status

To measure plant water status, leaf water potential (Ψ_w_) was determined on leaves at about 10-day intervals starting at anthesis until late dent stage (from the beginning of June to the end of July) using leaf cutter thermocouple psychrometers (J.R.D. Merrill Specialty Equipment, Logan, UT, USA) at mid-day (1200–1300 h). A 5-mm diameter leaf disk was taken from the midpoint along the length of the leaf blade of a young and fully expanded leaf and placed in a leaf cutter thermocouple psychrometer. Samples were taken from four individual plants for each genotype per soil moisture treatment in four replications. The leaf cutter thermocouple psychrometers were placed in a water bath at 25 °C for 4 h. Outputs from the psychrometers were recorded by a PSYPRO data logger (WESCOR, Inc., Logan, UT, USA). Three Ψ_w_ readings were taken from each sample and the average of the 3 readings was calculated for each of the four samples per plot. 

#### 4.3.2. Photosynthetic Pigments

Chlorophyll and carotenoids contents were determined on the same leaves used for Ψ_w_. Leaf samples were collected, placed in plastic bags and kept in a cooler and then brought to the laboratory. Two leaf discs with diameter of 10 mm each were taken from each leaf sample and placed in a vial containing 2 mL of absolute ethanol and incubated for 24 h at room temperature (25 °C) in the dark. Chlorophyll a (Chl a) and chlorophyll b (Chl b), and carotenoids were determined by measuring absorbance at 480, 645 and 663 nm using a spectrophotometer (Beckman Coulter DU 800 Spectrophotometer, Brea, CA, USA) and computed following the method of Hendry and Price [47]. Fifteen leaf discs with 16 mm diameter were also punched from the same leaf samples to determine specific leaf weight. The leaf discs were dried at 70 °C for 72 h and specific leaf weight was calculated as dry weight per unit leaf area.

#### 4.3.3. Heat Stress Measurements

Plants were grown in a greenhouse in 19 L pots containing Metro-Mix 200 (Sun Gro, Bellevue, WA, USA) in six replications under a 28 °C/23 °C day/night temperature regime and 16 h photoperiod. The corn seedlings were watered every day with a half strength Hoagland’s nutrient solution. Pots were rotated periodically to minimize position-induced plant-to-plant variation. When the plants were about five weeks old, temperature in the greenhouse was raised to 38 °C/33 °C day/night for seven days to impose heat stress on the plants. Before the heat stress treatment was imposed, initial (no heat stress) chlorophyll fluorescence measurements were made on young and fully expanded leaves. The leaves were dark adapted using dark-adaptation clips for 1 h and Fv/Fm (variable fluorescence/maximal fluorescence, maximum quantum efficiency of Photosystem II) was measured using an OS1-FL modulated chlorophyll fluorometer (Opti-Sciences, Hudson, NH). Chlorophyll fluorescence measurements were made on the heat stressed plants on the third, the fifth and the seventh day after the start of the heat stress treatment. Immediately after each Fv/Fm measurement, leaf discs were taken from the same leaf blade area that was used for Fv/Fm to determine chlorophyll content and cell membrane thermostability (CMT) (*in vivo* heat stress). CMT determination was made following the method of Sullivan [22,48]. Additional CMT determination was made on leaf samples collected from field plants which were not stressed. Heat stress was induced by incubating leaf discs (*in vitro*) at 40 °C for 6 h. Measurements on CMT were made at 1.5, 3.0, 4.5 and 6.0 h during the incubation period.

#### 4.3.4. Seed Composition Analysis

Corn kernels from each plot were sampled and analyzed for protein, oil, starch, and fiber percentage using a near-infrared (NIR) reflectance diode array feed analyzer (Perten, Springfield, IL, USA) [49,50]. Calibration equations were developed by Perten using Thermo Galactic Grams PLS IQ. The calibration curve has been regularly updated for unique samples according to AOAC methods [51,52].

### 4.4. Statistical Analysis

The data were analyzed using a two-way PROC ANOVA procedure in SAS ver. 9.2 (SAS Institute, Cary, NC, USA) to test genotypic differences under the two soil moisture treatments. Cell membrane thermostability and chlorophyll fluorescence data were analyzed using the PROC MIXED procedure in SAS to detect differences in measurements over time. 

## 5. Conclusion

In the present study, an effort was made to relate the level of aflatoxin contamination in kernels of corn genotypes to the physiological responses of the plants under drought and heat stress conditions. Reductions in maximum quantum efficiency of PS II, photosynthetic pigment content and cell membrane thermostabilty as well as leaf water potential and canopy temperature helped to evaluate the level of stress on the corn genotypes. The genotypes which were the most stressed, based on the physiological measurements, had the highest aflatoxin contamination and the least stressed had low aflatoxin contamination. Therefore, the present results suggest that there is a relationship between aflatoxin contamination in corn kernels and the physiological responses of the plants to drought and heat stress. These and other stress response-related physiological traits may help explain differences among corn genotypes in aflatoxin contamination. However, this did not apply to the aflatoxin resistant genotype, which was one of the most stressed genotypes but had the lowest level of aflatoxin contamination. This suggests that aflatoxin resistance in this genotype has more complex mechanisms, which may involve inhibition of fungal infection and growth or inhibition of aflatoxin biosynthesis after infection. 
